# Context-dependent variability in blue whale acoustic behaviour

**DOI:** 10.1098/rsos.180241

**Published:** 2018-08-08

**Authors:** Leah A. Lewis, John Calambokidis, Alison K. Stimpert, James Fahlbusch, Ari S. Friedlaender, Megan F. McKenna, Sarah L. Mesnick, Erin M. Oleson, Brandon L. Southall, Angela R. Szesciorka, Ana Širović

**Affiliations:** 1 Scripps Institution of Oceanography, University of California San Diego, 9500 Gilman Drive, La Jolla, CA 92093, USA; 2 Cascadia Research Collective, 218 ½ W 4th Ave., Olympia, WA 98501, USA; 3 Moss Landing Marine Laboratories, 8272 Moss Landing Road, Moss Landing, CA 95039, USA; 4 Institute for Marine Sciences, University of California Santa Cruz, 115 McAllister Way, Santa Cruz, CA 95064, USA; 5 Natural Sounds and Night Skies Division, National Park Service, 1201 Oakridge Drive, Fort Collins, CO 80525, USA; 6 Southwest Fisheries Science Center, National Marine Fisheries Service, NOAA, 8901 La Jolla Shores Drive, La Jolla, CA 92037, USA; 7 Pacific Islands Fisheries Science Center, National Marine Fisheries Service, NOAA, 1845 Wasp Blvd., Building 176, Honolulu, HI 96818, USA; 8 Southall Environmental Associates, 9099 Soquel Drive, Suite 8, Aptos, CA 95003, USA; 9 Texas A&M University Galveston, 200 Seawolf Parkway, Galveston, TX 77554, USA

**Keywords:** blue whale, *Balaenoptera musculus*, song, acoustic communication, behavioural context

## Abstract

Acoustic communication is an important aspect of reproductive, foraging and social behaviours for many marine species. Northeast Pacific blue whales (*Balaenoptera musculus*) produce three different call types—A, B and D calls. All may be produced as singular calls, but A and B calls also occur in phrases to form songs. To evaluate the behavioural context of singular call and phrase production in blue whales, the acoustic and dive profile data from tags deployed on individuals off southern California were assessed using generalized estimating equations. Only 22% of all deployments contained sounds attributed to the tagged animal. A larger proportion of tagged animals were female (47%) than male (13%), with 40% of unknown sex. Fifty per cent of tags deployed on males contained sounds attributed to the tagged whale, while only a few (5%) deployed on females did. Most calls were produced at shallow depths (less than 30 m). Repetitive phrasing (singing) and production of singular calls were most common during shallow, non-lunging dives, with the latter also common during surface behaviour. Higher sound production rates occurred during autumn than summer and they varied with time-of-day: singular call rates were higher at dawn and dusk, while phrase production rates were highest at dusk and night.

## 1. Introduction

Sound production is an important behavioural strategy for many species. The use of sound for reproductive purposes in terrestrial species has been well documented, for example, in songbirds [1–3], frogs [4,5], insects [6] and ungulates [7]. Many terrestrial species also commonly produce sounds associated with foraging [8] and other social interactions [9–12]. In the marine environment, where sound travels with little attenuation, sound production may play a critical role in many life functions. For instance, sound is important for invertebrates [13], fish [14–16] and marine mammal species [17–20]. Among marine mammals, it is often used for social and communicative purposes [18]. Baleen whales are very prolific producers of sounds [19,21,22]. The behavioural context of sound production has been examined for a subset of calls produced by well-studied baleen whale species, including the humpback whale (*Megaptera novaeangliae*) [23–26], the southern right whale (*Eubalena australis*) [27] and the North Atlantic right whale (*E. glacialis*) [28,29].

Blue whale (*Balaenoptera musculus*) sounds, particularly those produced by the northeast Pacific population, have also been extensively studied [30–34]. The acoustic repertoire of this blue whale population consists of three main sound types: A, B and D calls [30,35–38]. The pulsed A and tonal B sound types, each lasting approximately 15–20 s, can be produced individually at irregular intervals as singular calls [33] or together at regular intervals as A and B units within phrases. When repeated, these phrases form bouts of song and acoustically distinguish this population from other blue whale populations [31,39]. The A and B sound types have only been recorded from males and are thus considered to have a reproductive function, although this was based on a small sample of known callers [31,33]. These sounds are detected off southern California from June to January, and peak in September or October [40–43]. Blue whale D calls are shorter (less than 5 s), more frequency-modulated sounds that have been recorded from both males and females [30,31]. These variable down-swept calls appear to be commonly produced by different blue whale populations [44,45] and are probably used as social calls while foraging [33]. D calls are typically recorded in southern California from April to November, peaking during the summer [33,40,41].

Recent developments in bio-logging technology [46–49] have allowed for the collection of finer-scale data associated with sound production in a variety of marine mammals [50], including humpback whales [51,52], North Atlantic right whales [53], Antarctic minke whales [54], fin whales [55,56] and blue whales [33,57]. Multi-sensor tags, which are capable of recording acoustic and dive depth data as well as body movements, may allow for evaluation of the tagged whale's behaviour during sound production if a reliable method is available for determining which sounds are produced from the tagged animal. This analysis can be particularly difficult for low-frequency baleen whale sounds [50,55]. If sounds can be attributed to the tagged whale, the detailed behaviour of tagged whales producing sounds may also be compared to that of whales not producing sounds while tagged, to examine differences in behaviour. Ultimately, if long-term passive acoustic data are to be used to estimate whale distributions and densities [58–60], the behavioural context(s) of sound production must be understood.

Previous studies into the behavioural context of sound production in blue whales have been limited either in sample size, with a small number of tag deployments resulting in relatively few hours of collected data [57], or in the number of sounds detected, due to natural variation in sound production by any individual tagged whale [33]. Furthermore, because the recording durations of these tags are inherently restricted by memory, battery capacity and attachment method [50], only a small subset of data collected from blue whales was recorded at night [33,57], providing us with limited understanding of how blue whale behaviour varies between day and night. However, recent development of alternative attachment methods have resulted in longer deployments and have thus enhanced our ability to obtain behavioural and acoustic data from tagged blue whales for durations ranging from several days to weeks [61].

In this study, we evaluated how call and phrase production rates varied with respect to behavioural state, location, season and time-of-day in blue whales tagged off southern California. Our dataset included the acoustic and dive depth data collected from acoustic tags deployed on individuals over the course of 14 years, including data recorded during several long-duration deployments. This analysis provides the most extensive analysis into the behavioural context of blue whale calling in this region available to date.

## 2. Methods

### 2.1. Tag data collection

For these analyses we used data collected by tags deployed on blue whales off southern California from 2002 to 2016 (electronic supplementary material, table S1). The whales were tagged as part of multiple research efforts, including collaborations between the Cascadia Research Collective (CRC) and Scripps Institution of Oceanography (SIO), and during the Southern California Behavioural Response Study (SOCAL-BRS) [62]. All tag deployments were conducted in accordance with the ethical standards under CRC's Institutional Animal Care and Use Committee protocols (AUP-6), and under National Marine Fisheries Service (NMFS) permits as detailed in the Animal Ethics section. No additional permissions were needed to carry out fieldwork. Tag deployments used in this study include the deployments from southern California used in an earlier analysis of the behavioural context of blue whale calling [33]. Three types of sound- and movement-recording tags were deployed on blue whales: Bioacoustic Probes (Bprobes), Acousondes (both developed by Greeneridge Sciences, Inc.) and Dtags [63]. Bprobes are capable of sampling acoustic data at rates up to 20 kHz and are equipped with ancillary sensors for recording temperature, pressure, and, in versions produced after 2003, 2-axis acceleration. Accelerometer data enable the derivation of instantaneous body orientation of the whale during a dive cycle [64]. In addition to the auxiliary sensors found in the Bprobe, Acousondes contain an updated 3-axis accelerometer, a compass and the ability to collect higher-frequency acoustic data. The sampling capabilities of Dtags are similar to those of Acousondes, as they record depth, accelerometer and acoustic data, although they each had different hydrophone sensitivities [63]. The sampling rates for acoustic, auxiliary and accelerometer data varied with tag type and deployment (electronic supplementary material, table S1). Across all deployments, sampling rates were in the range of 1024–240 000 Hz for acoustic data, 1–800 Hz for accelerometer data and 1–50 Hz for auxiliary data (electronic supplementary material, table S1).

**Figure 1.**
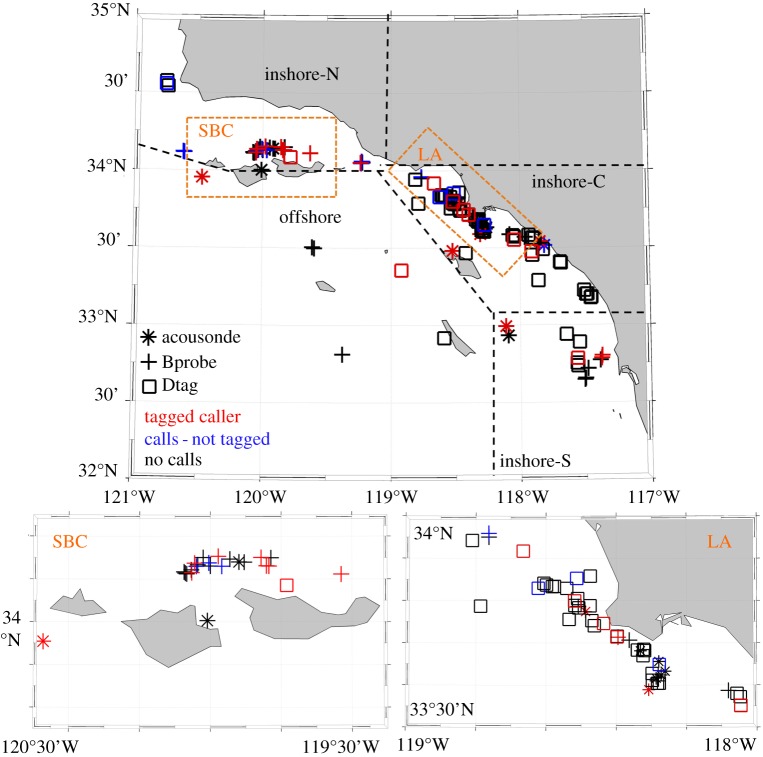
Locations of tagging events for all Acousondes (stars), Bprobes (crosses) and Dtags (squares) deployed on blue whales off southern California during 2002–2016. Tags that contained sounds that were attributed to the tagged whale are marked in red, while tags that contained sounds not assigned to the tagged whale are marked in blue. Tags that did not contain any sounds are plotted in black. The four areas used to classify tag deployment locations for statistical analyses are marked with bold, black-hashed lines. Two smaller areas that contained high densities of tag deployments (within the Santa Barbara Channel and off Los Angeles and Long Beach, CA) are marked in orange and shown as inset maps on the bottom.

Tags were deployed on blue whales in multiple regions [33]; however, we focused our analysis only on deployments conducted off southern California (figure 1) during ship-based efforts and shore-based tagging operations. Blue whales were tagged opportunistically, typically based on the ability to locate and track them visually. When an individual was chosen for tagging, the whale was approached using a rigid-hull inflatable boat (RHIB) and a tag was deployed using a metal or fibreglass pole. For the majority of deployments, the tag was attached to the whale with suction cups. However, starting in 2016, longer-duration tag attachment methods (darts instead of suction cups) were used [61]. When feasible, photo identification and biopsy samples were also collected from tagged whales.

Upon tag retrieval, digital data were downloaded from the tag to a computer. Only deployments with at least 15 min of high-quality acoustic data were included in this analysis [33]. As part of the SOCAL-BRS, some of the animals included in this analysis were exposed to simulated Navy sonar or pseudo-random noise [62,65,66] (electronic supplementary material, table S1); however, in those cases, data from during the exposure and 3 h after the exposure were excluded from analysis to eliminate potential impact of the exposure on the tagged whale's natural behaviour. Only acoustic and pressure data were used for this study because 3-axis accelerometer data were not collected on 44 of the 121 deployments available for analysis (they were only available for Acousonde and Dtag deployments).

### 2.2. Acoustic data analysis

We reviewed the acoustic data collected from all Bprobe and Acousonde deployments in spectrogram form in *Triton*, a Matlab-based (www.mathworks.com) software package [67]. We manually detected all blue whale A, B and D sounds based on visual and aural inspection of the spectrogram (calculated with 1 Hz and 0.1 s resolutions, using a Hanning window), and logged the start and end times of each sound. The acoustic data collected using Dtag deployments were decimated to a 600 Hz sample rate before plotting the spectrogram using custom Matlab software (calculated with 1.17 Hz and 0.02 s time resolution, using a Hamming window) and manually analysed for blue whale sounds, also logging start times of each sound.

When studying the behavioural context of sound production, it is important to note whether the tagged animal produced each sound recorded on the tag or not. However, there is no standard method for accomplishing this task. Previous studies have relied upon universal signal-to-noise ratio (SNR) cut-offs in order to assign sounds to tagged humpback whales [68], while, more recently, accelerometer data have been used to determine caller identity in tagged fin whales [55,56]. In general, the use of source-level estimates to confirm caller identity is problematic because tag placement on the animal can vary both within and between deployments, and because the anatomical location of the blue whale sound production mechanism remains unknown [69,70]. We could not rely on accelerometer data because they were not available for many deployments and, additionally, there has been no evidence to indicate that the same procedure used for fin whales will work for blue whales or for tag types other than Dtags. Furthermore, while the assignment of sounds to an individual is critical for many studies, our behavioural analysis is focused on evaluating the broader behavioural contexts associated with sound production, and the precise assignment of sounds to the tagged individual is less crucial. Therefore, by using a combination of high relative root-mean-square received levels (RL_rms_) and SNRs to attribute sound production to the tagged whale, we feel confident that the sounds that were used for analysis in this study were either produced by the tagged whale or a whale that was close to the tag, at approximately the same depth and possibly engaging in the same behaviour [71], though not necessarily the same sex. Tagged blue whales in this study were generally single or closely paired with another individual (electronic supplementary material, table S1).

We calculated the RL_rms_ and the SNR for each detected sound according to the following processes. We applied an infinite impulse response (IIR) bandpass filter to the data, with the bandpass frequencies based on the call type (A, B or D) and corresponding to the band of peak frequency for each call. The three frequency bands were 70–100 Hz for A calls, 38–55 Hz for B calls and 25–100 Hz for D calls. We used the third harmonic of B calls and the higher-frequency pulsed harmonics of A calls because these were louder and simpler to discern from low-frequency flow noise. We calculated RL_rms_ over the duration of each sound, where the duration was calculated based on 90% of the energy of the sound [29]. We also measured noise levels in 500 ms intervals during the 10 s prior to each call, and subtracted the lowest of these noise-level measurements from the RL_rms_ of the call to determine the SNR [68]. As transfer functions were not known for all tags, we calculated only relative values, and compared them to other relative values within a given deployment or for the same tag. We did not compensate for the built-in high-pass filters in the Dtag hardware, nor did we correct for other system sensitivities of any tag types. We performed all calculations in Matlab (www.mathworks.com).

To attribute sound production to the tagged (or nearby) whale as opposed to distant animals, we calculated the mean and standard deviations of RL_rms_ and SNRs for each call type and deployment. If the RL_rms_ or the SNR of an individual call was higher than the mean minus one standard deviation calculated for that deployment, we assigned the call to the tagged whale, otherwise we removed it from further analysis. For deployments that contained only one or two calls of one call type, we used the mean and standard deviations of RL_rms_ and SNRs calculated for all deployments of the same particular tag. In all other cases, this method resulted in at least some calls being assigned to the tagged whale on every deployment where calls were recorded. However, the method did reliably exclude very faint calls that were probably produced by distant animals. For our purposes, we use both ‘produced’ and ‘attributed to tagged whale’ as shorthand and in reference to sounds that were presumed to be produced by the tagged individual or nearby whale as determined based on these methods.

As we were interested in acoustic behaviour, we evaluated whether any individual A or B sound was produced as a singular call or as a unit within a phrase. First, we sorted all A and B detections based on the logged start time. We calculated inter-call intervals, measured as the time from the start of one call to the start of the next, for all A and B sounds. We classified all blue whale A and B detections that were not produced in a pattern with regular inter-call intervals as singular calls rather than phrase units [41]. We defined phrases as sequences of A and B calls where the start of one unit was followed by another within 49 ± 10 s for A–B units and within 51 ± 14 s for B–B units [40]. We defined repetitive phrases as sequences of AB phrases where the interval between the ending B unit of one phrase and the leading A unit of the next was 70 ± 29 s [40]. We grouped all single AB phrases, i.e. those that were neither preceded nor followed by another phrase, and repetitive phrases together into the same ‘phrase’ category. We also grouped them regardless of phrase composition (i.e. the ratio of A to B units within the phrase). We classified all D call detections as singular D calls.

### 2.3. Pressure data analysis and behavioural classification

We used pressure (i.e. depth) data as a proxy for the behavioural state of the tagged blue whale. The pressure data from each deployment was loaded into the Acq*Knowledge* software (v. 3.9.1, Biopac Systems, Inc.), and individual dives were identified based on changes in pressure over time. A dive was defined as a submergence that exceeded 10 m in depth. For each recorded dive, we marked the following dive characteristics using the program's manual selection tools: dive start time (in local time; defined as the start time of submergence from the surface); dive duration (defined as the time between submergence from and re-emergence to the surface); maximum dive depth (defined as the maximum depth reached during the dive); time spent at bottom of dive (defined as the time between the whale's descent from and ascent to the surface, and based on the depth change over time); and the number of vertical lunges present within a dive. Although vertical as well as horizontal lunges can be easily identified using accelerometer data [64,72], our dive analyses were limited to the use of pressure data because accelerometer data were not available for all deployments. Thus, for our study, only vertical lunges were identified, and these were defined as vertical excursions in excess of 5 m from the bottom of a dive [57].

If singular A, singular B or D calls, or phrases attributed to the tagged blue whale were detected within a dive, we recorded the number and type of sounds present. We determined the depth of production for each sound based on pressure data and the time at the start of the sound. Many of the Bprobe and Acousonde deployments exhibited systematic offsets in depth measured at the surface. To correct this, we calculated the average surface depth for each deployment and when it differed from 0 m, we applied this value as a correction factor to all sound production depths. We excluded dives during which the tag fell off (i.e. the final dive of each deployment) from all analyses.

We used a combination of the maximum dive depth and the presence of vertical lunges detected within a dive to broadly classify each dive into one of five behavioural states: shallow non-lunging, shallow lunging, deep non-lunging, deep lunging and surface behaviour. We classified all dives without vertical lunges that did not exceed the 50 m depth as shallow, non-lunging dives [33]. Dives that exceeded the 50 m depth without vertical lunges were classified as deep, non-lunging dives. We classified all dives that did not exceed the 50 m depth but contained vertical lunges as shallow lunging dives, and all dives that contained vertical lunges at a depth exceeding 50 m as deep lunging dives. If the tagged blue whale spent extended time near the surface, without any identifiable diving behaviour, we classified this behaviour as surface behaviour. Bouts of surface behaviour were distinguishable from normal surface breath intervals based on the duration of time spent within 5 m of the surface (generally in excess of 10 min). For these behavioural categories, we use lunging and non-lunging as shorthand to describe the presence or absence of vertical lunges only; no other changes in the tagged whale's orientation were recorded because 3-axis accelerometer data were not available on all deployments. Furthermore, although lunges are generally indicative of foraging [57,64,72], we cannot exclude that feeding occurred also during non-lunging states because we did not analyse available accelerometer data (for reasons noted above). As we were primarily focused on identifying broad behavioural contexts, our behavioural state classifications should not be considered as a precise evaluation of a tagged whale's feeding or non-feeding behaviour. Similarly, because our dive analyses were limited to changes in pressure data over time, we could not evaluate whether bouts of surface behaviour included feeding events.

### 2.4. Sound production and behavioural context analysis

To statistically evaluate the effect of location, season, behavioural state and time-of-day on sound production, we modelled the occurrence of singular A, singular B, and D calls, and AB phrases using generalized estimating equations (GEEs) [73]. We used this approach because GEEs allow for estimates of population average parameters from correlated or clustered data [73]. Thus, by clustering the data into units based on individual deployments, we were able to account for differences between individual tagged whales as well as autocorrelation within an individual deployment.

We classified deployments based upon the location (latitude and longitude) of the initial tagging event into one of four groups: inshore-south (south of 33° N, In-S), inshore-central (between 33° and 34° N, In-C), inshore-north (north of 34° N, In-N) and offshore (offshore of Santa Catalina Island and south of the Channel Islands) (figure 1). Seasonal trends in call and phrase production rates were evaluated by classifying data based on month of initial deployment: spring, for deployments occurring between March and May; summer, for deployments occurring from June to August; and autumn, for tags deployed between September and November. However, because just one deployment occurred during the spring (electronic supplementary material, table S1), seasonal tests were conducted between summer and autumn only. Diel patterns in call and phrase production rates were similarly analysed by classifying data into four time-of-day periods: dawn, day, dusk and night. We used the definitions of these periods as described by Wiggins *et al*. [32], based on times of nautical twilight, sunrise and sunset at the location each tag was deployed. We binned all data based on the average dive duration into 12 min intervals. If a 12 min bin crossed between two time-of-day periods, we classified the bin into one of the four categories based on the majority of minutes spent in a particular period. We defined the behavioural state for each bin based on the five dive categories described previously. If multiple diving behaviours were recorded within a 12 min interval, we classified the behavioural state as the behaviour that took up the majority of time. Finally, the number of singular A, singular B and D calls, and phrases attributed to the tagged whale were counted over each 12 min interval. Hourly call and phrase production rates were then calculated and used as the response variable in the GEE, and location, season, behavioural state and time-of-day were used as covariates. Mean hourly call and phrase production rates per behavioural state were also calculated for each tagged caller, and mean production rates across all tagged callers were then plotted across the five behavioural states.

We used individual whales as the clustering unit for the GEE, and modelled the call rate using Poisson distribution and log-link function with an autoregressive correlation structure to account for temporal correlation between bins within a single deployment. We used the standard robust sandwich variance estimate for all reported results [73]. We performed all analyses using the geeglm() function of the ‘geepack’ package [71,74] in the R Studio (v. 1.0.153) statistical software platform [75].

## 3. Results

A total of 874.1 h of acoustic and dive profile data collected from 121 tags (mean attachment duration = 7.2 h; s.d. = 18.0 h; median = 2.2 h) deployed on blue whales off southern California were analysed (electronic supplementary material, table S1). A total of 13 individuals were tagged more than once but none of those tags contained calls attributable to the tagged animal. Of all tag deployments, 22.3% (27 tags) contained sounds that were attributed to the tagged whale or the tagged whale's group, an additional 12.4% (15 tags) contained blue whale sounds that were not attributed to the tagged whale and 65.3% (79 tags) contained no blue whale sounds (figure 1; electronic supplementary material, table S1). Among all tagged animals, 13% (16 whales) were male, 47% (57 whales) were female, for 5% (six whales) sex could not be determined from the available sample and 35% (42 blue whales) were without skin samples for sex determination (electronic supplementary material, table S1). Within the 16 tags deployed on male blue whales (electronic supplementary material, table S1), 50% (eight tags) contained sounds attributed to the tagged individual, 25% (four tags) contained sounds that were not attributed to the tagged whale and 25% (four tags) contained no blue whale sounds. For the 56 tags that were deployed on females (electronic supplementary material, table S1), 5% (three tags) contained sounds attributed to the tagged individual, 13% (seven tags) contained sounds that were not attributed to the tagged whale and 82% (46 tags) contained no blue whale sounds.

Overall, out of the total of 4514 blue whale sounds detected from 42 acoustic records, 73% (3308) were attributed to 27 tagged individuals. The majority of all sounds attributed to tagged individuals were phrases (880; comprising 880 A units and 1361 B units) and D calls (550). Similar numbers of singular A and B calls were produced (229 and 288, respectively). The majority of singular A calls (117) and singular B calls (175) were produced around the same time as phrases, albeit at longer and more irregular intervals than A and B phrase units. Sounds attributed to tagged male blue whales consisted predominately of phrases (81; comprising 81 A units and 85 B units), singular A calls (74) and singular B calls (25), although D calls (131) were also produced in high numbers, especially by one male tagged in 2010 (with 117 attributed D calls) (electronic supplementary material, table S1). Sounds attributed to female blue whales, on the other hand, were fewer and consisted of eight singular A calls, one singular B call and 24 D calls (electronic supplementary material, table S1). However, all of the singular A and B calls, as well as one D call, were recorded by tags deployed on three females that were all tagged while in close association with another blue whale. Furthermore, the RL_rms_ and SNR values calculated for these calls were relatively low in comparison to calls of the same type recorded during other tag deployments. Therefore, it is possible that these calls may have been produced by the nearby, associated whales rather than the tagged females. The remaining 23 D calls were recorded on a tag deployed on a female in a tightly associated pair whose sex was determined based on being sighted with a calf in a previous year (electronic supplementary material, table S1). Finally, we should note that the majority (354) of D calls were produced by a single individual of unknown sex.

**Figure 2.**
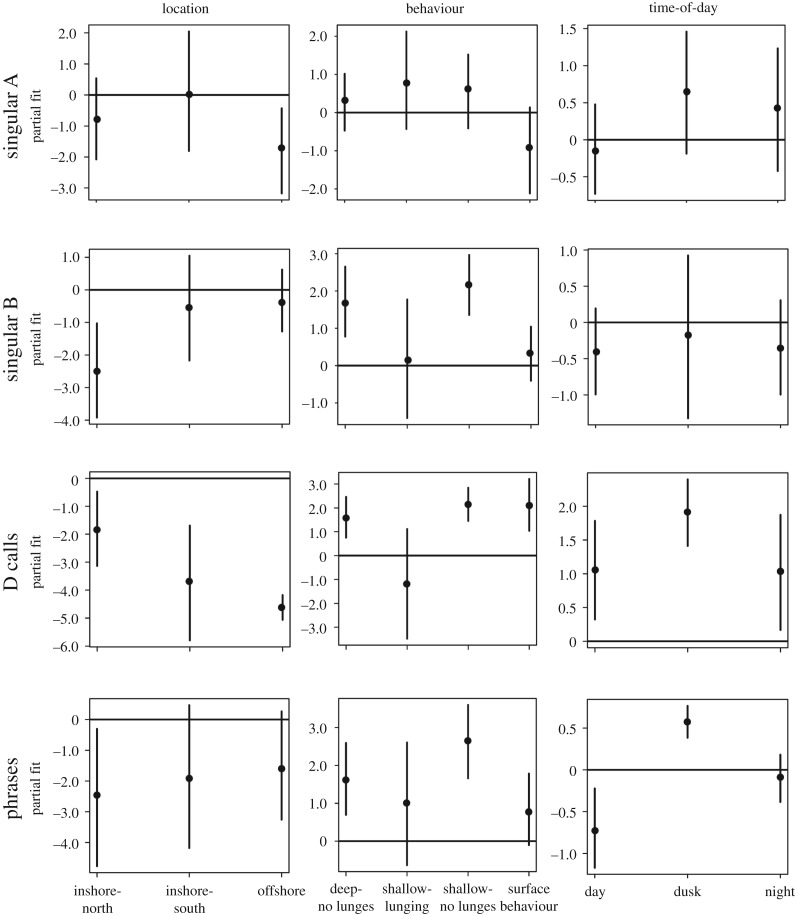
Partial fit plots output from the generalized estimating equations (GEEs) used to model blue whale call and phrase production rates as a function of location, behavioural state and time-of-day. The horizontal lines at zero in each plot represent the reference level of each factor: inshore-central for location; deep lunging dives for behavioural state; and dawn for time-of-day.

Most tag deployments, and the largest number of acoustic records containing calls, occurred within the inshore-central and inshore-north locations (figure 1), particularly off Los Angeles/Long Beach and within the Santa Barbara Channel. However, the effect of location on call and phrase production rates varied with sound type (figure 2, table 1). Singular B call and phrase production rates recorded from blue whales tagged in the inshore-north region were significantly lower than the rates from individuals tagged in other regions (B calls: *p* = 0.001, phrases: *p* = 0.043) (figure 2, table 1). Singular A call rates were significantly lower in the offshore region than in any other tagging region (*p* = 0.010) (figure 2, table 1). D call rates were higher from blue whales tagged in the inshore central region than in any other region (figure 2, table 1).

**Table 1.** Results from the generalized estimating equations (GEEs) used to assess spatial, temporal and behavioural variability in singular call and phrase production rates. For each sound type, coefficient parameter estimates (Cp. est), robust standard errors (s.e.) and *p*-values for each of the levels within the four factor variables (location, season, behavioural state and time-of-day (TOD)) are presented. Variables and associated levels with significant *p*-values are marked with an asterisk and italicized. Statistics are based upon the difference from the reference level of each factor: inshore-central for location; autumn for season; deep lunging dives for behavioural state; and dawn for time-of-day.

sound type	factor	level	Cp. est	s.e.	*p*-value
singular A	*location*	In-N	−0.786	0.702	0.2630
		In-S	−0.020	0.922	0.983
		*Off*	−*1.738*	*0.686*	0.010*
	*season*	*summer*	−*1.399*	*0.543*	0.010*
	behaviour	Dp-no lunges	0.309	0.392	0.431
		Sh-lunges	0.818	0.657	0.213
		Sh-no lunges	0.596	0.505	0.238
		surface	−0.935	0.579	0.106
	TOD	day	−0.152	0.320	0.636
		dusk	0.621	0.431	0.150
		night	0.410	0.420	0.329
singular B	*location*	*In-N*	−*2.552*	*0.785*	0.001*
		In-S	−0.575	0.869	0.508
		Off	−0.337	0.504	0.504
	season	summer	−1.271	0.678	0.061
	*behaviour*	*Dp-no lunges*	*1.660*	*0.501*	0.001*
		Sh-lunges	0.171	0.805	0.832
		*Sh-no lunges*	*2.131*	*0.415*	*2.90* × *10*^−7^*
		surface	0.343	0.363	0.344
	TOD	day	−0.404	0.320	0.207
		dusk	−0.189	0.591	0.748
		night	−0.358	0.344	0.297
D calls	*location*	*In-N*	−*1.965*	*0.731*	0.007*
		*In-S*	−*3.155*	*0.882*	*3.50* × *10*^−4^*
		*Off*	−*4.793*	*0.248*	*<2.00* × *10*^−16^*
	*season*	*summer*	−*1.731*	*0.776*	0.026*
	*behaviour*	*Dp-no lunges*	*0.869*	*0.358*	0.015*
		Sh-lunges	−0.161	0.35	0.645
		*Sh-no lunges*	*1.122*	*0.428*	0.009*
		*surface*	*1.524*	*0.464*	0.001*
	*TOD*	day	0.239	0.191	0.21
		*dusk*	*1.202*	*0.279*	*1.60* × *10*^−5^*
		night	0.471	0.328	0.15
phrases	*location*	*In-N*	−*2.460*	*1.217*	0.043*
		In-S	−1.884	1.231	0.126
		Off	−1.566	0.880	0.075
	season	summer	−0.180	0.870	0.836
	*behaviour*	*Dp-no lunges*	*1.656*	*0.467*	*3.90* × *10*^−4^*
		Sh-lunges	1.037	0.849	0.222
		*Sh-no lunges*	*2.652*	*0.490*	*6.20* × *10*^−8^*
		surface	0.826	0.471	0.079
	*TOD*	*day*	−*0.719*	*0.234*	0.002*
		*dusk*	*0.583*	*0.100*	*6.80* × *10*^−9^*
		night	−0.086	0.138	0.532

**Figure 3.**
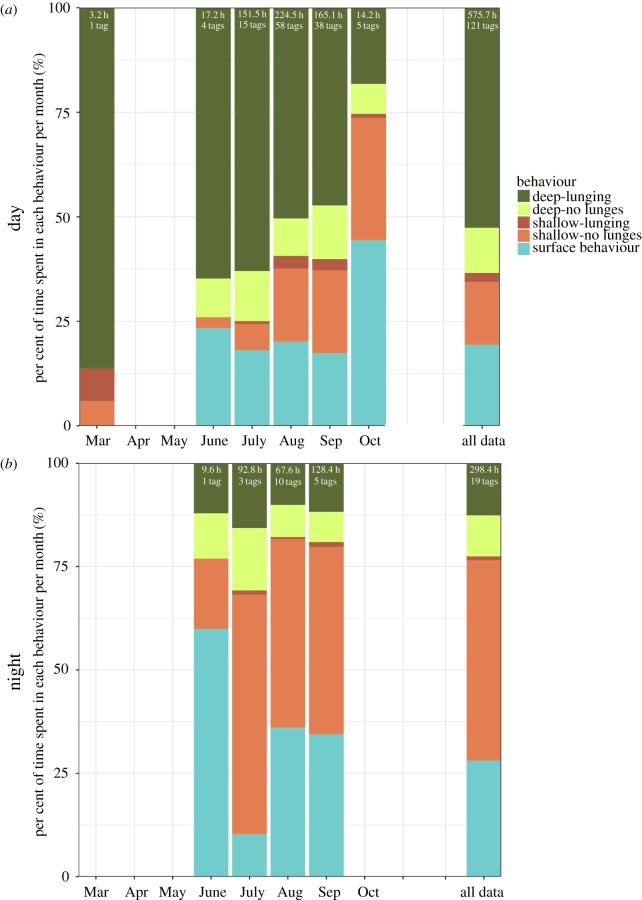
The per cent of time tagged blue whales spent in each behavioural state during the day (*a*) and night (*b*) per month. The number of tags containing day- or night-time data, and the total hours of data available during each diel period are also listed per month. No tags were deployed on blue whales off southern California during the months of April or May.

Deep lunging dives comprised the majority of daytime tag data, while shallow non-lunging dives and surface behaviour dominated at night (figure 3). However, the proportion of time spent within these behavioural states differed by month (figure 3). Specifically, during the day, the per cent of time spent in deep lunging dives decreased between the summer and autumn, while shallow non-lunging and surface behaviours increased (figure 3). With the exception of the single March deployment, tagged blue whales spent the least amount of time in the shallow lunging dive state each month (figure 3).

**Figure 4.**
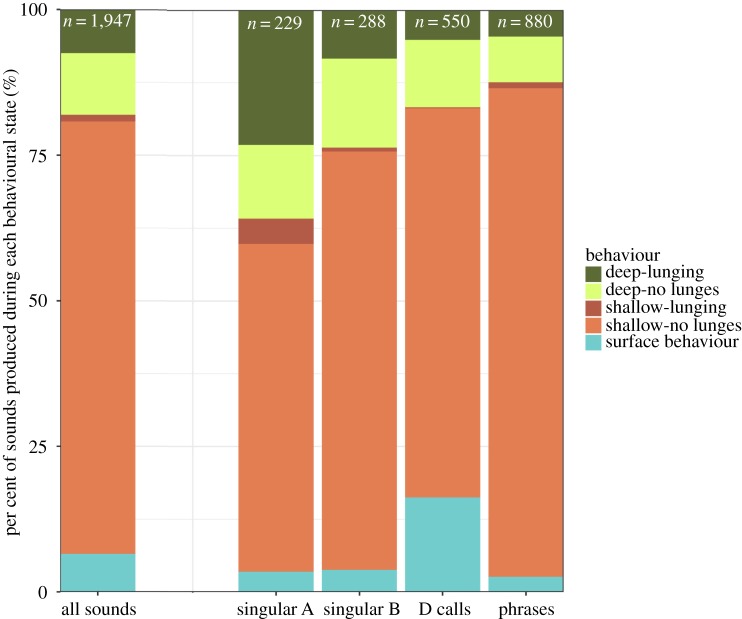
The per cent of singular A, singular B and D calls, and phrases that were produced within different behavioural states. The total number of detections for each sound type are listed, with each count of phrases possibly combining multiple A and B units. Only sounds that were attributed to tagged blue whales are included.

Blue whale call and phrase production rates recorded during the autumn were higher than those recorded during the summer, though only differences in singular A and D call production rates were significant (A calls: *p* = 0.010, D calls: *p* = 0.026) (table 1). This seasonal variation may correspond to the increased per cent of time that blue whales spent within shallow non-lunging and surface behavioural states during the autumn (figure 3), because we observed increased call and phrase production by blue whales within these behavioural states. About 75% of all sounds attributed to tagged individuals were produced during shallow, non-lunging dives (figure 4), and another 10% were produced at shallow depths during deep non-lunging dives. The fewest number of sounds were produced during shallow lunging dives (around 1% of all sounds; figure 4). In comparison to the proportion of time that blue whales spent in deep lunging and non-lunging dives overall (figure 3), the number of sounds produced within these behavioural states were far fewer than the number of sounds produced during shallow lunging and surface behavioural states (figure 4).

**Figure 5.**
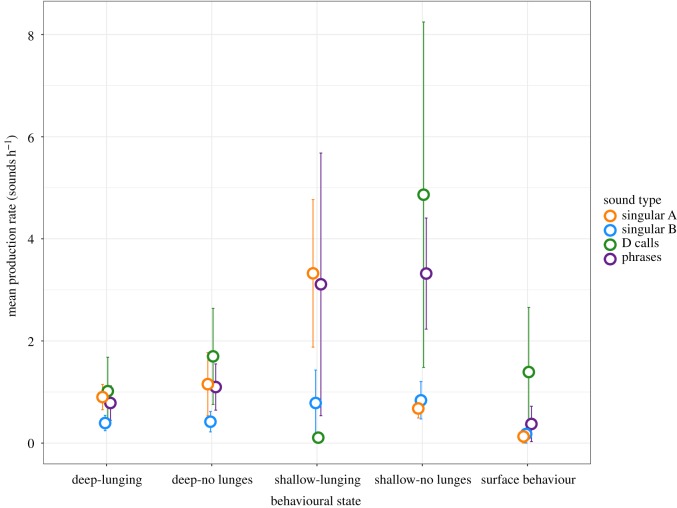
Mean hourly production rates of singular A (orange), singular B (blue) and D calls (green), and phrases (purple) within different behavioural states. Bars represent the standard error of the mean, calculated for each sound type. Only data from tag deployments containing calls attributed to the tagged whale are included. Production rates were calculated using only calls that were assigned to the tagged individuals.

**Figure 6.**
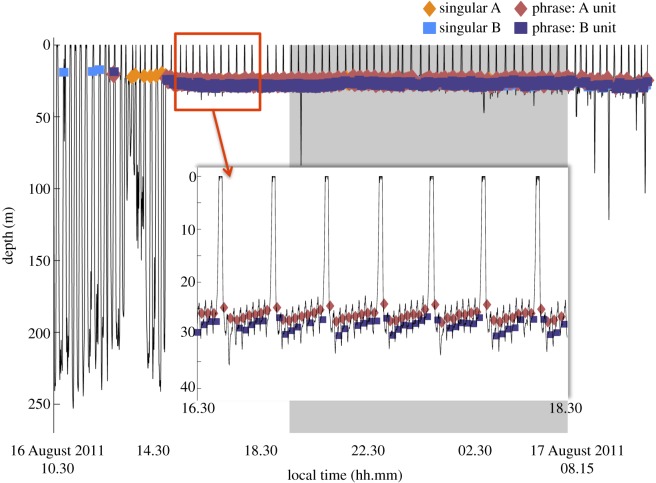
Blue whale dive profile recorded from an Acousonde deployed on 16 August 2011, showing extended singing behaviour. Two hours of the repetitive phrase, or song, bout are shown in the inset. Night is shaded in grey. Only sounds attributed to the tagged individual are included.

**Figure 7.**
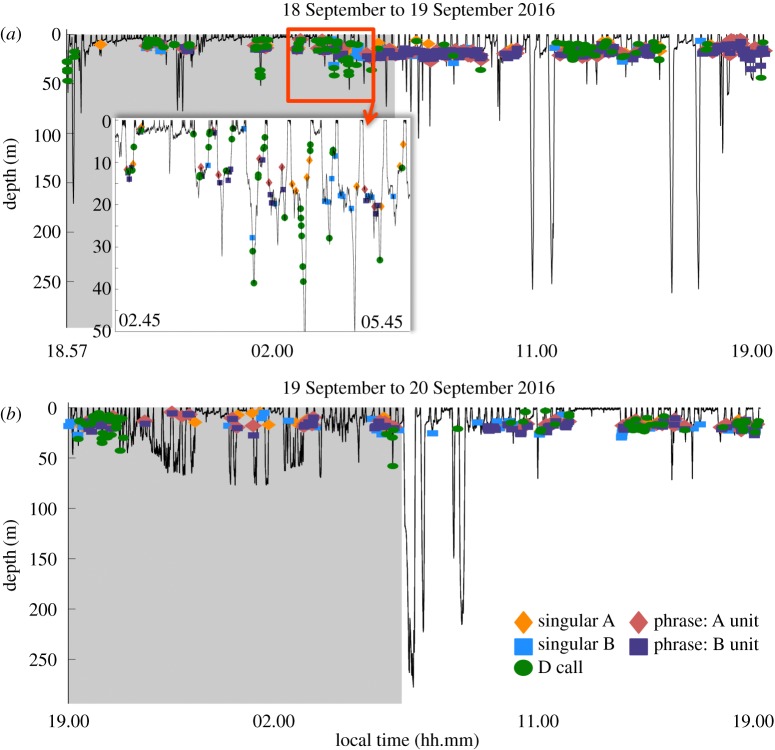
Blue whale dive profile recorded from an Acousonde deployed on 18 September 2016, showing variability between the behaviours associated with singular call and phrase production. Only 48 h from the full 5 d deployment are shown: (*a*) the first 24 h from 18 September to 19 September 2016; and (*b*) 19 September to 20 September 2016. Three hours from 19/09/2016 are highlighted in the inset to illustrate behaviour during singular call and single phrase production. Night is shaded in grey. Only sounds attributed to the tagged individual are included.

Phrase production rates in particular were significantly higher during shallow non-lunging and deep non-lunging dives than during other dive behaviours (figures 2 and 5, table 1). During the production of A and B units within phrases, dives were consistently shallower (less than 35 m in maximum depth) than during the production of singular calls (figure 6). Apart from surface breath intervals, blue whales producing repetitive phrases, or singing (*n* = 11 individuals), often regularly dived to a relatively consistent depth for each dive over a period of hours, displaying a similar behaviour throughout the duration of the song bout (figure 6). During these bouts, singing individuals also consistently ended each dive with a B unit before surfacing (figure 6). Blue whale behaviours exhibited during the production of single phrases, those neither preceded nor followed by another phrase, were less consistent (figures 6 and 7). In some cases, single phrases were produced towards the end or beginning of deeper dives by an individual that would later begin singing (figure 6), while during other deployments, the tagged blue whale produced both single phrases and singular calls within a short time frame (figure 7).

**Figure 8.**
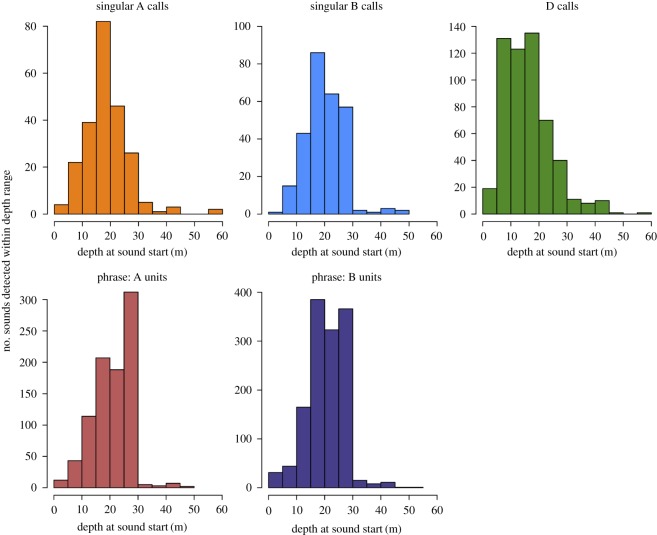
Histograms of depth at the start of sound production for all singular A, singular B, D calls, and phrase units attributed to tagged individuals. Note different *y*-axis scales.

During the production of singular A, B and D calls, blue whale behaviour was more variable (figure 7), with dives frequently extending beyond 35 m, although call production generally still occurred within the upper 30 m (figures 7 and 8). Both singular B and D call rates were higher during non-lunging dives than during lunging dives (figures 2, 4 and 5, table 1). D call rates were also higher during extended surface behaviour than during lunging dives (figures 2, 4 and 5, table 1), and D call production rates were the highest of any sound type during these surface bouts (figures 2, 4 and 5, table 1). Differences in singular A call production rates between different behavioural states were insignificant (figures 2 and 5, table 1). However, A calls were produced in higher numbers than other sound types during shallow lunging dives (figure 4).

Regardless of dive behaviour, the majority of all blue whale sounds attributed to tagged animals were produced in the top 30 m of the water column (figure 8). Both singular A and B calls most frequently occurred between 15 and 20 m. When produced as parts of a phrase, A and B units were most commonly produced between 25 and 30 m, but they were also frequently produced between 15 and 20 m. In general, D calls were regularly produced at shallow depths ranging from 5 to 20 m.

Time-of-day had a significant impact on blue whale call and phrase production rates. In general, the production rates of all sound types showed crepuscular increases compared to other time-of-day periods. Singular A call rates were lowest during the day and highest at dusk, but differences were not significant (figure 2, table 1). Singular B call rates, on the other hand, were lower during the day and at night than during dawn or dusk, but differences were not significant (figure 2, table 1). D call production rates were significantly higher at dusk and similar across the other three periods (figure 2, table 1). Phrase production rates were also highest at dusk, but significantly lower during the day than during the night or at dawn (figure 2, table 1).

## 4. Discussion

We observed significant variability in blue whale call and phrase production rates with respect to different behavioural states, in addition to spatial and temporal patterns, and across sexes. The majority of all sounds attributed to tagged blue whales were produced at shallow depths (less than 30 m) during shallow non-lunging dives. However, there were distinct differences between the behaviours associated with the production of singular calls versus phrases. Singular A, B and D calls were more frequently produced during non-lunging dives or during bouts of surface behaviour, and phrases were typically produced during shallow non-lunging dives. Furthermore, while the large majority of the tagged blue whales in this study were females, very few of those tags had sounds attributed to them (5%), while the attribution of sounds to tagged male blue whales (50%) was much more common.

The consistent behaviour exhibited during the production of repetitive phrases suggests an exclusive behavioural state for blue whales. Similar to the observations made by Stimpert *et al.* [56] on fin whales, tagged blue whales that were producing repetitive phrases, or singing, repeatedly made long, stereotypically ‘U-shaped dives’ to a consistent shallow depth, often for extended periods of time. In general, the behaviours associated with song production in male blue whales have been described as solitary and travelling, not feeding [33]. When foraging, blue whales exhibit lunge-feeding behaviours [64,72,76,77], often exploiting prey patches at depth [33,78]. The absence of dives containing vertical lunges during song bouts, coupled with the shallow production depths of repetitive song phrases, supports the hypothesis that feeding and singing behaviours in blue whales may be mutually exclusive states [32,33,41,79]. As song has only been recorded from male blue whales and is thought to be associated with reproduction [31,33], the repetitive production of phrases to form long song bouts in the absence of foraging could also be used as an indicator of the singing male's condition to potential mates [80–82].

Contrary to the consistent diving behaviour exhibited by singing blue whales, the behaviours that we observed from tagged individuals producing singular calls were much more variable, which suggests that singular A and/or B calls may have a distinct behavioural purpose from A and B sounds produced as units within phrases. Blue whales in both the Pacific and Atlantic have been shown to exhibit different behaviours when producing singular calls versus singing, with the former more frequently engaging in feeding, milling and resting, and the latter in travelling behaviours [33,83]. Additionally, the infrequent production of A and/or B calls may be used by males to maintain pair bonds during feeding [33]. The use of foraging-associated calls has been well documented in many bird and mammalian species, and in several cases, these calls may be used for reproductive benefits [8]. For example, Krunkelsven *et al.* [84] observed that captive male bonobos used food-associated calls to attract females to a food source, which ultimately led to copulations between the females and calling males.

Although singular A and B calls were previously only recorded from male blue whales [33], we also recorded singular A and B calls on tags deployed on three female individuals (electronic supplementary material, table S1). The depths at which these singular calls were recorded were similar to those observed in other deployments, and the tagged females exhibited no unusual diving behaviour. While numerous examples exist in insect and frog species of females producing similar signals to those produced by males [4,85,86], focal follow data collected during and after each of these deployments indicate that all three females were tagged while interacting closely with another whale as part of a pair (electronic supplementary material, table S1). Although these calls were attributed to the tagged females based on our methods, the calculated RL_rms_ and SNR values were lower than the majority of other singular calls. Therefore, it is possible that the recorded sounds could have been produced by the nearby, untagged individuals of unknown sex rather than the tagged females.

We found production rates of D calls, which have previously been associated with foraging [33,41], to be significantly higher during shallow non-lunging dives and periods of surface behaviour than during other dive types. This increase in D calling at shallower depths during non-lunging dives, coupled with the fact that D calls were apparently recorded from both males and females [33], suggests that D calls may be used in multiple contexts, although we reiterate that our methods did not allow for evaluation of whether bouts of surface behaviour included feeding events, so foraging could have been occurring during surface behaviour. Many species may use food-associated calls as broadcast advertisements [8], but most mammals and birds produce such calls at reduced amplitudes, durations and rates compared to social calls [87]. Blue whale D calls, which are both shorter in duration and produced less frequently than A or B calls, also have a reduced propagation range compared to the propagation capability of B calls [31,88,89]. Thus, D calls are probably used as shorter-range social calls between nearby individuals. However, it is important to point out that a single blue whale tag deployment from September 2016 contained approximately 66% (364 of 550) of all D calls (figure 8; electronic supplementary material, table S1). This particular whale spent extended amounts of time at or near the surface (22.9 out of 102.5 h of data collected), so it is possible that the observed differences in D call production rates may be largely driven by this individual's behaviour. Additionally, although D calls have been attributed to female blue whales in previous studies [33], we only recorded D calls on two tags deployed on females (electronic supplementary material, table S1). This difference could be due to differences in study locations, because our analysis included only data collected from individuals tagged off southern California and many of them near shore, or to the assigning of calls to the tagged animal. Alternatively, this could also imply that females do not produce sounds very often during the summer, when most tag data were collected.

Based on these data, we suggest that there are potential behavioural advantages for sound production at shallower depths. Despite differences observed between dive type and behavioural state during the production of singular calls and phrases, tagged blue whales produced the majority of sounds at shallow depths, generally within 30 m of the surface. This depth range is consistent with the average depths of blue whale calling (20–30 m) that Oleson *et al.* [33] reported using a subset of these data, and is also similar to, albeit a bit deeper than, the average depths of fin whale calling (10–15 m) recorded by Stimpert *et al.* [56] from tag deployments off southern California. Additionally, our results are consistent with the average depth of B call production proposed in a theoretical model of blue whale sound production by Aroyan *et al.* [70]. Oleson *et al.* [33] speculated that signal output may be maximized at these shallow calling depths. In their model of fin whale calling, Weirathmueller *et al.* [90] show that fin whale 20 Hz pulses increased in amplitude and range when produced between 25–30 m, as well as between 65–70 m. For male blue whales that are seeking mates or producing male–male displays [91] and thus singing for extended periods of time, the ability to communicate over longer ranges with minimal energy expenditure would be advantageous. The gradual changes in dive depths during the production of repetitive song phrases observed in the data collected from one individual (figure 6), which were not associated with any systematic data drifts, may illustrate an inadvertent upward drift of the animal while singing and could be related to body condition [92]. Similar dive drifts have also been linked to foraging and the ratio of fat to lean body mass in elephant seals [93]. This drift was not noted in other whales diving to similar depths. Although details regarding the sound production mechanism in baleen whales remain largely unknown, a recent model presented by Dziak *et al*. [69] suggests a pulsed-air mechanism for B call production in blue whales that is anatomically similar to the model proposed for humpback whales [94]. Simulations run on humpback whale songs recorded off Hawaii suggest that male humpbacks may select the optimum singing depth and frequency in order to maximize propagation potential [95]. The consistent behaviour that we recorded from tagged individuals during bouts of song production certainly indicates that blue whales may be achieving the same benefit by producing calls at these particular depths.

The seasonal and diel differences that we observed in blue whale sound production rates may be correlated to temporal changes in behaviour. Production rates of all sound types were higher during the autumn than during the summer, although only differences between singular A and D call rates were significant. The production of sounds by tagged individuals during each month of tag deployments, with the exception of March, is similar to the occurrence of B calls reported from long-term studies [41,96]. In contrast to the summer peak in D calling recorded in these studies [33,41], D call production rates in our data were greater during the autumn. Our analysis also showed significant trends in blue whale call production rates with respect to time-of-day: singular B and D call rates were highest at dusk, while singular A call rates increased both at dawn and dusk. These patterns are similar to the B calling peaks reported during twilight hours [32]. Blue whale phrase production rates in our dataset were also highest at dusk and significantly lower during the day than at night or dawn. As blue whales typically prey upon diel-migrating euphausiids, which are found in high concentrations at depth during the day [76,77,97,98], individuals may time periods of singing behaviour to coincide with periods of reduced prey availability [32,33]. Similar behavioural trade-offs have been observed during the day in European robins [99,100], and in nightingales [101], who adjust their nightly singing rates at dusk according to the energy reserves they have accumulated during the day.

The proportion of time blue whales spent in different non-acoustic behavioural states also shifts seasonally. The amount of time that tagged blue whales spent in deep diving states, specifically deep lunging dives indicative of foraging [33,76–78], was generally greatest during the summer months, between June and August. The single blue whale tagged in March of 2015 also primarily exhibited deep and shallow lunging dive behaviours. However, shallow non-lunging behaviours, which might be more commonly associated with song production [33], comprised the majority of all hours of data collected during the autumn, between September and October. In many mammalian species, including elephants [102], pinnipeds [103] and ungulates [104–107], the limitation or cessation of food consumption by males during the breeding season is common—the motivation for which appears to be the reduction of foraging time in favour of breeding efforts. While it has previously been accepted that both sexes of several baleen whale species also limit food intake during their annual migrations to breeding grounds [89], blue whales may exhibit both foraging and reproductive behaviours on their low-latitude breeding grounds [108,109]. Therefore, it is also possible that multiple behavioural states may also be exhibited at feeding sites. Although southern California is primarily considered to be a seasonal feeding ground for blue whales, our analysis indicates a temporal separation between two behavioural states for blue whales in this region: individuals may begin feeding as early as spring, continuing through summer until the autumn, at which time reproductive behaviours begin to dominate. Unfortunately, little-to-no data were collected from late autumn to spring, so it is difficult to assess whether these behavioural trends extend to adjacent months and seasons.

We recorded significant behavioural differences between calling and singing blue whales, in addition to both spatial and temporal patterns in call and phrase production rates, but also discovered several substantial biases in tagging effort. First and foremost, although the overall sex ratio of the Northeast Pacific population of blue whales is estimated to be about even, our data show a clear bias towards tagging of female blue whales, because 72% (57 out of 79) of the individuals for which sex determination occurred were females. In addition, the majority (95%) of all tag deployments occurred inshore of the Channel Islands. In mammals, males generally disperse more than females [110–113], while females of many species are typically more social [114]. Our sex bias in tag deployments off southern California suggests that female blue whales may spend more time inshore, and are thus more likely to be encountered, compared to males. Furthermore, the ability to tag blue whales varies with respect to the individual whale's behavioural state: it is much easier to deploy a tag on an animal that is resting or milling than on an animal that is travelling quickly. Because deployment requires a close approach to an individual, how different sexes respond to close approach could bias towards a particular sex. Previous studies on blue whales off southern California have observed both slow, shallow diving behaviour in females [31] as well as increased singing from solitary males travelling offshore [33]. Therefore, it is possible that the tagging bias arose not only from differential use of habitat by male and female blue whales in this area but also from sexual differences in behaviour or approachability.

In addition to the sex bias that we discovered in tag deployments, the data analysed in our study were collected from opportunistic tagging efforts that occurred primarily between July and September. Therefore, the autumn peak in D call production in our dataset may be due to the paucity of data collected during late-spring and early-summer deployments, which is when D calls have been shown to increase in other studies [33,40,41]. However, approximately 87% (481 out of 550) of all D calls were produced by two blue whales (eight total individuals produced D calls) tagged in September of 2010 and September of 2016, so the increase in D call production rates during the autumn is biased by the behaviour of these two individuals. Furthermore, over the years of tag deployments, different survey efforts were targeting animals in specific behavioural states, or under different environmental conditions and geographical locations for different studies. Therefore, our collection of tag data cannot be considered a truly random sample of the population. Overall, understanding these biases is critical to allowing us to better understand the behavioural context of sound production rates, which is needed for better interpretation of the ecology and habitat use of blue whales from passive acoustic data.

The data collected through the use of multi-sensor tags can allow for the assessment of the behavioural context of sound production. However, there are several limitations associated with studying blue whale acoustic behaviour through tag deployments. Most importantly, the assignment of any recorded sound to the tagged individual rather than a nearby whale is not a straightforward task. Recent studies on fin whales have indicated that caller identity can be confirmed based on detection of sounds in accelerometer data [55,56]; however, this method is only applicable to tags capable of recording high-sample-rate accelerometer data and, further, has proved less successful for the longer-duration and higher-frequency sounds produced by blue whales [115]. Owing to these issues, and to standardize analysis among our dataset that included a large proportion of tags without high-frequency three-dimensional accelerometer sampling (36%), we assigned recorded sounds to tagged animals based on calculated relative RL_rms_ and SNR for all sounds detected within a given tag deployment. Based on these methods, it is possible that some of the sounds that we attributed to tagged individuals may have been produced by another whale swimming nearby, particularly for whales associated in pairs as is typical for blue whales; however, if this were the case, it is possible that the adjacent whale was engaging in the same general behaviour as the tagged individual [116]. In addition, based on our methods, deployments containing many calls that were all relatively faint would still have had calls attributed to the tagged individual. An alternative approach would have been to use a universal RL_rms_ or SNR cut-off to attribute calls to tagged blue whales; however, our data were complicated by the use of multiple tag types, each with its own unique system specifications, so RL_rms_ levels were often not comparable. We chose not to use a universal SNR cut-off because the SNR also varies by tag type and deployment given differences in hardware configuration. Thus, because we cannot say with absolute certainty that tagged individuals produced all of the calls attributed to them, our reported call and phrase production rates should not be considered absolute production rates from any particular individual. However, we are confident that the broad behavioural contexts associated with calling in this analysis give insight into general blue whale acoustic behaviour across sexes and behavioural states.

There are a number of threats facing the northeast Pacific population of blue whales, particularly ship strikes and anthropogenic noise [65,117–119]. Understanding baseline behaviours of this population is critical to being able to evaluate possible impacts of anthropogenic activity to this and other animal populations. Although the sound types produced by blue whales in this area have been well described [31–33,39,79], still relatively little is known about the behaviours associated with sound production. Overall, our analysis provides valuable insight into how blue whale acoustic behaviour varies on different temporal, spatial and, most importantly, behavioural scales. Understanding this variability and establishing a baseline for the behaviours, in addition to recognizing the biases and limitations associated with the use of tag data, is critical to allowing us to better understand the behavioural context of sound production. Such a baseline will, in turn, allow for better interpretation of the ecology and habitat use of blue whales from passive acoustic data and will provide a broader context for interpretation of data from studies on the impact of human activities on this population.

## Supplementary Material

Supplemental Table: Tag Deployment Details

## Supplementary Material

Blue whale dive profile analyses

## Supplementary Material

Blue whale call detections and determinations data

## Supplementary Material

Blue whale GEE - R code

## Data accessibility

The datasets supporting this article have been uploaded as part of the electronic supplementary material.
