# Supplementary material for: Context-dependent variability in blue whale acoustic behaviour
Source: R Soc Open Sci. 2018 Aug 8;5(8):180241. doi: 10.1098/rsos.180241 (PMC6124089; doi:10.1098/rsos.180241)
Supplement: Supplemental Table: Tag Deployment Details [file rsos180241supp1.docx]

Supplemental Table S1. Deployment details for all tags deployed on blue whales between 2002—2016 with data used in this study, including: deployment date; tag on time; tag type; latitude (Lat), longitude (Long), and location of tagging event (Loc); sampling rates (in Hz) for Acoustic, Aux (pressure) and Accel (accelerometer) data (Note that Bprobe deployments had either no (na) or only 2-dimensional accelerometer data); Data (h) = number of hours of acoustic and dive profile data analyzed from each deployment; types of Sounds attributed to the tagged individual; the Sex of the tagged individual; and # in Grp, the number of whales associated in a group, including the tagged animal. Unique whale ID identifier is noted, when known. Data collected during Dtag deployments that included controlled exposure experiments are marked with either one or two asterisks, depending on whether the exposure consisted of simulated Navy sonar or pseudo-random noise, respectively. In those cases, data from during and 3-hr after the exposure were excluded from analysis to eliminate any potential impact of the exposure on the whale’s natural behaviour, and the number of hours of analyzed data listed has been updated to reflect this. Acousonde deployments that used dart rather than suction cup attachment methods are indicated with an asterisk. Deployments that contained A, B, or D calls or phrases that were assigned to the tagged whale are shaded in gray, and the types of sounds are indicated. Deployments during which blue whale sounds were detected, but none were assigned to the tagged individual are marked with an asterisk. For sex determination, M: male, F: female, U: sex could not be determined; na: skin sample not yet analyzed; and ns: no skin sample collected.

| **Tagging event information** | | | | | | **Sampling rates (Hz)** | | | **Data analysis** | | | | **Whale ID** |
| --- | --- | --- | --- | --- | --- | --- | --- | --- | --- | --- | --- | --- | --- |
| **Date** | **Time on (PDT)** | **Tag type** | **Lat (N)** | **Long (W)** | **Loc** | **Acoustic** | **Aux** | **Accel** | **Data (h)** | **Sounds** | **Sex** | **# in Grp** |  |
| 6/26/02 | 9:03 | Bprobe | 34°06.85 | 120°04.25 | In-N | 5,461 | 1 | na | 2.8 | none | U | 1 | 1864 |
| 6/27/02 | 10:49 | Bprobe | 34°06.92 | 120°03.17 | In-N | 2,520 | 1 | na | 1.3 | D's | ns | 1 |  |
| 6/30/02 | 15:49 | Bprobe | 32°47.10 | 117°22.63 | In-S | 2,520 | 1 | na | 15 | A's, B's, Phrases | M | 1 | 336 |
| 7/24/03 | 11:17 | Bprobe | 33°30.25 | 119°36.72 | Off | 2,520 | 1 | 1 | 1.5 | none | F | 1 | 1941 |
| 7/24/03 | 15:45 | Bprobe | 33°29.81 | 119°35.80 | Off | 2,048 | 1 | 1 | 0.8 | none | ns | 1 | 2000 |
| 8/22/03 | 15:48 | Bprobe | 32°48.68 | 119°22.50 | Off | 2,048 | 1 | 1 | 0.7 | none | F | 1 | 2024 |
| 7/20/04 | 14:17 | Bprobe | 34°07.31 | 120°03.25 | In-N | 1,024 | 1 | 1 | 0.6 | none | ns | 1 |  |
| 7/21/04 | 13:09 | Bprobe | 34°06.74 | 120°04.01 | In-N | 1,024 | 1 | 1 | 0.9 | none | U | 1 | 1295? |
| 8/22/06 | 12:00 | Bprobe | 32°39.34 | 117°30.10 | In-S | 1,024 | 1 | 1 | 6.5 | none | F | 1 | 2239 |
| 8/23/06 | 12:47 | Bprobe | 32°43.08 | 117°28.90 | In-S | 1,024 | 1 | 1 | 1.2 | none | F | 1 | 2283 |
| 8/24/06 | 15:30 | Bprobe | 32°48.07 | 117°22.38 | In-S | 1,024 | 1 | 1 | 3.9 | A's, B's, Phrases | M | 1 | 1132 |
| 8/25/06 | 12:33 | Bprobe | 32°38.85 | 117°30.40 | In-S | 1,024 | 1 | 1 | 0.6 | none | F | 1 | 2302 |
| 9/6/07 | 10:01 | Bprobe | 34°07.88 | 119°50.50 | In-N | 2,048 | 1 | 1 | 4.4 | A's, D | ?^[[1]](#footnote-1)^ | 2 | 1811 |
| 9/6/07 | 10:24 | Bprobe | 34°07.83 | 119°50.07 | In-N | 2,048 | 1 | 1 | 1 | A's, D | F | 2 | 442 |
| 9/6/07 | 12:40 | Bprobe | 34°08.39 | 119°54.25 | In-N | 2,048 | 1 | 1 | 0.8 | none | U | 1 |  |
| 9/6/07 | 16:03 | Bprobe | 34°09.21 | 119°58.67 | In-N | 2,048 | 1 | 1 | 11.5 | A's, B's, Phrases | M | 2 | 306 |
| 9/6/07 | 16:45 | Bprobe | 34°07.82 | 119°58.07 | In-N | 2,048 | 1 | 1 | 0.6 | none* | U | 2 |  |
| 9/7/07 | 9:50 | Bprobe | 34°09.04 | 120°01.22 | In-N | 2,048 | 1 | 1 | 0.9 | none | M | 2 | 1571 |
| 9/7/07 | 18:14 | Bprobe | 34°07.02 | 120°04.47 | In-N | 2,048 | 1 | 1 | 1.7 | none | ns | 1 | 2495 |
| 9/8/07 | 9:07 | Bprobe | 34°08.01 | 120°02.14 | In-N | 2,048 | 1 | 1 | 0.8 | none | ?^[[2]](#footnote-2)^ | 2 | 302 |
| 9/8/07 | 9:45 | Bprobe | 34°07.80 | 120°02.39 | In-N | 2,048 | 1 | 1 | 2.4 | none* | ns | 2 |  |
| 9/8/07 | 10:02 | Bprobe | 34°08.24 | 120°02.68 | In-N | 2,048 | 1 | 1 | 5.9 | A | na | 2 | 2097 |
| 6/14/08 | 10:17 | Bprobe | 32°46.30 | 117°23.21 | In-S | 2,048 | 1 | 1 | 7.7 | none | F | 1 | 636 |
| 8/14/08 | 14:32 | Bprobe | 34°09.05 | 119°51.38 | In-N | 2,048 | 1 | 1 | 1.6 | A | F | 2 | 1250 |
| 8/16/08 | 10:44 | Bprobe | 34°06.70 | 119°37.8 | In-N | 2,048 | 1 | 1 | 10.6 | A's, B | F | 2 | 536 |
| 9/9/08 | 13:38 | Bprobe | 33°57.54 | 118°45.81 | In-C | 2,048 | 1 | 1 | 1.8 | none* | F | 2 | 2549 |
| 9/9/08 | 14:05 | Bprobe | 33°57.02 | 118°45.78 | In-C | 2,048 | 1 | 1 | 0.4 | none | F | 2 | 1305 |
| 9/10/08 | 14:37 | Bprobe | 34°08.82 | 119°56.69 | In-N | 2,048 | 1 | 1 | 0.5 | none | F | 2 | 953 |
| 9/12/08 | 14:17 | Bprobe | 34°07.84 | 120°00.01 | In-N | 2,048 | 1 | 1 | 0.3 | none | M | 2 | 1697 |
| 9/12/08 | 14:30 | Bprobe | 34°08.28 | 120°00.27 | In-N | 2,048 | 1 | 1 | 1.3 | none* | F | 1 | 1539 |
| 8/3/09 | 14:00 | Bprobe | 34°09.00 | 119°49.74 | In-N | 2,048 | 1 | 1 | 4.2 | none | M | 1 | 2621 |
| 8/4/09 | 14:03 | Bprobe | 34°03.07 | 119°14.18 | In-N | 2,048 | 1 | 1 | 2.9 | A's, B's, Phrases | M | 2 | 1224 |
| 8/4/09 | 17:25 | Bprobe | 34°02.89 | 119°13.88 | In-N | 2,048 | 1 | 1 | 0.6 | none* | F | 2 | 794 |
| 8/5/09 | 10:45 | Bprobe | 34°03.56 | 119°13.66 | In-N | 2,048 | 1 | 1 | 4.5 | none* | F | 1 | 2565 |
| 9/16/09 | 16:43 | Bprobe | 34°03.22 | 119°13.39 | In-N | 2,048 | 1 | 1 | 7 | none | F | 2 | 667 |
| 9/21/09 | 16:10 | Bprobe | 34°07.14 | 120°37.11 | In-N | 2,048 | 1 | 1 | 0.8 | none* | F | 2 | 946 |
| 9/21/09 | 16:30 | Bprobe | 34°06.99 | 120°36.67 | In-N | 2,048 | 1 | 1 | 1 | none* | ?^[[3]](#footnote-3)^ | 2 | 483 |
| 8/17/10 | 8:44 | Dtag-v2 | 33°11.87 | 117°29.52 | In-C | 64,000 | 50 | 50 | 8.8 | none | F | 1 | 667 |
| 8/17/10 | 8:48 | Dtag-v2 | 33°13.37 | 117°30.46 | In-C | 64,000 | 50 | 50 | 6.3 | none | F | 1 | 1993 |
| 8/18/10 | 7:50 | Dtag-v2 | 33°11.93 | 117°28.96 | In-C | 64,000 | 50 | 50 | 6.7 | none | F | 1 | 2243 |
| 8/23/10 | 9:14 | Dtag-v2* | 33°33.86 | 117°54.18 | In-C | 96,000 | 50 | 50 | 2.5 | none | F | 1 | 2152 |
| 8/23/10 | 9:20 | Dtag-v2* | 33°33.54 | 117°53.16 | In-C | 64,000 | 50 | 50 | 2.9 | none | F | 1 | 1216 |
| 8/23/10 | 14:10 | Bprobe | 33°35.22 | 118°04.84 | In-C | 8,192 | 1 | 1 | 1.2 | none | F | 1 | 1750 |
| 8/23/10 | 15:44 | Bprobe | 33°35.22 | 118°04.84 | In-C | 8,192 | 1 | 1 | 1.5 | none | F | 1 | 1750 |
| 8/26/10 | 9:25 | Bprobe | 33°34.18 | 117°57.80 | In-C | 8,192 | 1 | 1 | 5.1 | none | na | 1 | 1341 |
| 8/27/10 | 10:25 | Dtag-v2* | 33°34.31 | 118°02.83 | In-C | 96,000 | 50 | 50 | 4.3 | none | F | 1 | 1758 |
| 8/28/10 | 8:15 | Dtag-v2* | 33°41.09 | 118°19.33 | In-C | 96,000 | 50 | 50 | 30.9 | none | F | 1 | 646 |
| 8/28/10 | 8:21 | Dtag-v2* | 33°40.90 | 118°19.14 | In-C | 96,000 | 50 | 50 | 1.4 | none | F | 3 | 961 |
| 8/29/10 | 9:27 | Bprobe | 33°42.40 | 118°21.77 | In-C | 8,192 | 1 | 1 | 1.5 | none* | F | 1 | 794 |
| 8/29/10 | 10:22 | Bprobe | 33°42.82 | 118°23.69 | In-C | 8,192 | 1 | 1 | 2.4 | A's, B's, Phrases | M | 1 | 1376 |
| 8/31/10 | 9:28 | Bprobe | 33°47.16 | 118°30.47 | In-C | 8,192 | 1 | 1 | 10.6 | none | F | 1 | 2152 |
| 8/31/10 | 10:37 | Dtag-v2** | 33°47.20 | 118°47.20 | In-C | 96,000 | 50 | 50 | 5 | none | F | 1 | 2779 |
| 9/1/10 | 14:30 | Dtag-v2 | 33°49.99 | 118°34.92 | In-C | 96,000 | 50 | 50 | 0.7 | none | ?^[[4]](#footnote-4)^ | 2 | 992 |
| 9/1/10 | 15:59 | Dtag-v2** | 33°49.76 | 118°37.33 | In-C | 96,000 | 50 | 50 | 1.4 | none* | M | 2 | 2215 |
| 9/1/10 | 16:13 | Dtag-v2** | 33°49.75 | 118°32.43 | In-C | 96,000 | 50 | 50 | 1.2 | none | F | 2 | 634 |
| 9/2/10 | 12:38 | Dtag-v2** | 33°50.02 | 118°34.54 | In-C | 96,000 | 50 | 50 | 2.1 | none | F | 1 | 2779 |
| 9/3/10 | 12:49 | Dtag-v2* | 33°50.46 | 118°36.36 | In-C | 96,000 | 50 | 50 | 1.4 | none | M | 2 | 678 |
| 9/3/10 | 12:53 | Dtag-v2* | 33°50.21 | 118°35.90 | In-C | 96,000 | 50 | 50 | 1.3 | none | F | 1 | 634 |
| 9/4/10 | 13:03 | Dtag-v2** | 33°45.16 | 118°28.15 | In-C | 96,000 | 50 | 50 | 0.7 | none | F | 2 | 2744 |
| 9/8/10 | 13:20 | Dtag-v2** | 34°05.12 | 119°47.06 | In-N | 96,000 | 50 | 50 | 1 | A's, B's, Phrases | U | 2 | 913 |
| 9/22/10 | 10:47 | Dtag-v2* | 33°47.96 | 118°31.09 | In-C | 96,000 | 50 | 50 | 2.6 | D's | M | 1 | 2681 |
| 9/22/10 | 11:15 | Dtag-v2 | 33°47.31 | 118°31.05 | In-C | 96,000 | 50 | 50 | 1 | none | F | 1 | 2752 |
| 9/22/10 | 15:54 | Acousonde | 33°46.44 | 118°29.23 | In-C | 25,811 | 10 | 10 | 14.9 | A's, B's, Phrases | U | 1 | 853 |
| 9/23/10 | 14:56 | Dtag-v2** | 33°42.94 | 118°23.82 | In-C | 96,000 | 50 | 50 | 1.6 | none | F | 2 | 2756 |
| 7/29/11 | 11:17 | Dtag-v2* | 34°32.40 | 120°44.61 | In-N | 96,000 | 50 | 50 | 4.1 | none | F | 1 | 2095 |
| 7/29/11 | 12:29 | Dtag-v2* | 34°33.22 | 120°45.42 | In-N | 192,000 | 50 | 50 | 2.9 | none* | M | 1 | 20 |
| 7/30/11 | 9:09 | Dtag-v2** | 34°34.36 | 120°45.29 | In-N | 192,000 | 50 | 50 | 2 | none | F | 2 | 2237 |
| 8/1/11 | 9:54 | Dtag-v2* | 33°48.29 | 118°30.58 | In-C | 192,000 | 50 | 50 | 2.9 | none | F | 2 | 544 |
| 8/2/11 | 8:32 | Dtag-v2 | 33°51.48 | 118°28.42 | In-C | 96,000 | 50 | 50 | 1.1 | none | F | 2 | 794 |
| 8/2/11 | 9:02 | Dtag-v2** | 33°51.16 | 118°30.74 | In-C | 96,000 | 50 | 50 | 2.3 | none* | M | 2 | 20 |
| 8/3/11 | 11:19 | Dtag-v2 | 33°34.93 | 117°55.96 | In-C | 96,000 | 50 | 50 | 1.2 | none | F | 1 | 139 |
| 8/3/11 | 12:39 | Dtag-v3 | 33°34.89 | 117°56.58 | In-C | 96,000 | 50 | 50 | 1.4 | none* | M | 1 | 506 |
| 8/6/11 | 16:24 | Dtag-v2** | 33°10.77 | 117°27.11 | In-C | 96,000 | 50 | 50 | 1.3 | none | F | 1 | 39 |
| 8/6/11 | 16:44 | Dtag-v2** | 33°10.46 | 117°27.43 | In-C | 96,000 | 50 | 50 | 1.7 | none | F | 1 | 763 |
| 8/7/11 | 14:00 | Dtag-v2 | 32°44.12 | 117°33.58 | In-S | 96,000 | 50 | 50 | 0.8 | none | F | 1 | 2815 |
| 8/7/11 | 14:56 | Dtag-v2* | 32°45.18 | 117°33.85 | In-S | 192,000 | 50 | 50 | 3 | none | F | 1 | 2815 |
| 8/8/11 | 10:59 | Dtag-v2* | 32°47.01 | 117°33.71 | In-S | 96,000 | 50 | 50 | 4.8 | B's | M | 2 | 2851 |
| 8/8/11 | 14:38 | Dtag-v2* | 32°53.32 | 117°32.69 | In-S | 192,000 | 50 | 50 | 1.2 | none | F | 1 | 2852 |
| 8/9/11 | 11:50 | Dtag-v2** | 33°27.12 | 117°54.29 | In-C | 96,000 | 50 | 50 | 3.1 | none | F | 4 | 2857 |
| 8/9/11 | 13:46 | Dtag-v2** | 33°28.52 | 117°54.69 | In-C | 96,000 | 50 | 50 | 1.2 | A's | ?^[[5]](#footnote-5)^ | 2 | 2858 |
| 8/16/11 | 10:30 | Acousonde | 33°35.34 | 118°18.46 | In-C | 116,151 | 20 | 20 | 21.8 | A's, B's, Phrases | ns | 2 |  |
| 8/17/11 | 8:25 | Bprobe | 33°36.58 | 118°16.96 | In-C | 2,048 | 1 | 1 | 3.9 | none | F | 2 | 709 |
| 8/19/11 | 9:59 | Acousonde | 33°36.97 | 118°17.18 | In-C | 2,003 | 20 | 20 | 2.3 | none | ?^[[6]](#footnote-6)^ | 1 | 968 |
| 10/3/11 | 9:43 | Acousonde | 33°39.35 | 118°16.72 | In-C | 10,100 | 10 | 10 | 1.7 | none | F | 1 | 763 |
| 10/4/11 | 13:41 | Acousonde | 33°37.96 | 118°15.65 | In-C | 10,100 | 10 | 10 | 1.6 | none | F | 1 | 1142 |
| 10/4/11 | 15:01 | Acousonde | 33°37.42 | 118°16.33 | In-C | 10,100 | 10 | 10 | 0.6 | none | F | 1 | 600 |
| 8/4/12 | 12:50 | Dtag-v2** | 33°42.85 | 118°23.87 | In-C | 64,000 | 50 | 50 | 16.1 | D's | na | 2 | 2904 |
| 8/4/12 | 17:03 | Dtag-v3 | 33°44.76 | 118°26.14 | In-C | 64,000 | 50 | 50 | 2.2 | D's | ?^[[7]](#footnote-7)^ | 1 | 2905 |
| 8/27/12 | 13:35 | Acousonde | 33°32.45 | 117°50.83 | In-C | 4,005 | 20 | 20 | 8.7 | A's, Phrases | ?^[[8]](#footnote-8)^ | 2 | 1307 |
| 8/28/12 | 13:12 | Acousonde | 33°30.92 | 117°48.79 | In-C | 4,005 | 20 | 20 | 4 | none* | F | 2 | 1254 |
| 10/18/12 | 10:00 | Dtag-v2** | 33°21.27 | 118°55.14 | Off | 96,000 | 50 | 50 | 3.6 | A's, B's, Phrases | na | 1 | 2708 |
| 7/10/13 | 11:56 | Dtag-v3* | 33°24.05 | 117°41.12 | In-C | 240,000 | 20 | 200 | 1.4 | none | ?^[[9]](#footnote-9)^ | 2 | 667 |
| 7/12/13 | 7:33 | Dtag-v3* | 33°24.44 | 117°41.47 | In-C | 240,000 | 25 | 250 | 2.1 | none | na | 1 | 958 |
| 7/26/13 | 15:47 | Dtag-v3 | 33°38.92 | 118°16.64 | In-C | 240,000 | 50 | 500 | 3.4 | none* | ?^[[10]](#footnote-10)^ | 2 | 2212 |
| 8/2/13 | 10:15 | Dtag-v3 | 33°36.27 | 118°16.63 | In-C | 240,000 | 50 | 500 | 4.1 | none | na | 1 | 2262 |
| 8/2/13 | 10:26 | Dtag-v2 | 33°36.25 | 118°16.94 | In-C | 64,000 | 50 | 50 | 0.9 | none | F | 1 | 1216 |
| 8/2/13 | 14:53 | Dtag-v3 | 33°36.68 | 118°17.83 | In-C | 240,000 | 50 | 500 | 2.1 | none | na | 1 | 2262 |
| 8/5/13 | 8:30 | Dtag-v3* | 33°34.80 | 118°03.39 | In-C | 240,000 | 50 | 500 | 2.6 | none | na | 1 | 1074 |
| 9/16/13 | 10:15 | Dtag-v3* | 33°47.27 | 118°28.46 | In-C | 240,000 | 50 | 500 | 1.9 | none | na | 1 | 440 |
| 7/30/14 | 11:39 | Dtag-v3* | 33°45.33 | 118°32.03 | In-C | 240,000 | 25 | 250 | 1.8 | none | na | 2 | 1758 |
| 7/30/14 | 14:29 | Dtag-v3* | 33°46.98 | 118°30.39 | In-C | 240,000 | 25 | 250 | 1.4 | none | F | 1 | 2779 |
| 7/31/14 | 11:31 | Dtag-v3 | 33°55.03 | 118°39.89 | In-C | 240,000 | 25 | 250 | 26.4 | A's, B's, Phrases | ns | 1 | 1127 |
| 8/1/14 | 13:39 | Dtag-v3 | 33°56.53 | 118°48.63 | In-C | 240,000 | 25 | 250 | 4 | none | ns | 1 | 3071 |
| 8/5/14 | 10:52 | Dtag-v3 | 33°27.95 | 118°25.25 | In-C | 240,000 | 25 | 250 | 5.5 | none | na | 1 | 3072 |
| 8/6/14 | 8:34 | Dtag-v3* | 33°37.64 | 118°17.87 | In-C | 240,000 | 25 | 250 | 3.4 | none | na | 1 | 2294 |
| 8/6/14 | 10:22 | Dtag-v3 | 33°40.98 | 118°20.38 | In-C | 240,000 | 25 | 250 | 1.4 | none | F | 1 | 2610 |
| 8/26/14 | 13:25 | Acousonde | 33°40.87 | 118°19.88 | In-C | 25,811 | 40 | 800 | 3.1 | none | na | 1 | 2394 |
| 9/8/14 | 8:58 | Dtag-v3 | 33°44.45 | 118°27.72 | In-C | 240,000 | 25 | 250 | 8.5 | none | na | 2 | 3073 |
| 9/13/14 | 8:46 | Dtag-v3* | 33°40.17 | 118°19.42 | In-C | 240,000 | 25 | 250 | 2 | none | F | 1 | 139 |
| 9/18/14 | 8:50 | Dtag-v3 | 33°29.34 | 117°49.43 | In-C | 240,000 | 25 | 250 | 4.9 | none | na | 1 | 2617 |
| 9/19/14 | 8:49 | Dtag-v3* | 33°33.05 | 118°02.74 | In-C | 240,000 | 25 | 250 | 2.2 | A's, B | na | 2 | 2998 |
| 9/19/14 | 8:52 | Dtag-v3* | 33°33.10 | 118°02.73 | In-C | 240,000 | 25 | 250 | 2.2 | A's, B's, Phrases | M | 2 | 2056 |
| 3/17/15 | 12:10 | Dtag-v3 | 33°17.33 | 117°51.58 | In-C | 240,000 | 25 | 250 | 3.2 | none | na | 2 | 2702 |
| 8/20/15 | 13:35 | Dtag-v3 | 32°56.42 | 117°38.74 | In-S | 240,000 | 25 | 250 | 2 | none | na | 1 |  |
| 10/6/15 | 9:33 | Dtag-v3 | 32°54.99 | 118°35.32 | Off | 240,000 | 25 | 250 | 6.8 | none | ns | 1 |  |
| 7/16/16 | 12:12 | Acousonde* | 34°00.17 | 120°00.51 | In-N | 1,814 | 10 | 100 | 92.5 | none | na | 1 | 3090 |
| 7/17/16 | 9:28 | Acousonde* | 34°08.42 | 119°55.15 | In-N | 1,814 | 10 | 100 | 102.5 | none | na | 1 |  |
| 8/15/16 | 11:15 | Acousonde* | 32°56.02 | 118°05.56 | In-S | 12,226 | 10 | 100 | 3.9 | none | na | 1 |  |
| 8/17/16 | 13:28 | Acousonde* | 32°59.59 | 118°06.70 | In-S | 12,226 | 10 | 100 | 45.8 | D | ns | 1 |  |
| 9/18/16 | 13:15 | Acousonde* | 33°57.24 | 120°28.32 | Off | 12,226 | 10 | 100 | 96.8 | A's, B's, Phrases | na | 2 |  |
| 9/18/16 | 18:57 | Acousonde* | 33°28.85 | 118°31.43 | In-C | 12,226 | 10 | 100 | 102.6 | A's, B's, D's, Phrases | ns | 2 |  |

1. Likely M based on three positions as trailing individual in pair. [↑](#footnote-ref-1)
2. Likely M based on four positions as trailing individual in pair. [↑](#footnote-ref-2)
3. Likely F based on three positions as leading individual in pair. Also sighted as possible mother in two different years. [↑](#footnote-ref-3)
4. Likely F based on multiple positions as leading individual in pair, and was also marked as mother in a mother-calf sighting. However, analyzed skin and blubber samples that were attributed to the animal came back M. [↑](#footnote-ref-4)
5. Likely M based on two positions as trailing individual in same pair as deployment above on same day. [↑](#footnote-ref-5)
6. Likely F based on four positions as leading individual. [↑](#footnote-ref-6)
7. Likely F based on being sighted in a mother-calf pair. [↑](#footnote-ref-7)
8. Likely M based on three positions as trailing individual in pair. [↑](#footnote-ref-8)
9. Likely F based on three positions as leading individual in pair. [↑](#footnote-ref-9)
10. Likely M based on two positions as trailing individual in pair. [↑](#footnote-ref-10)
